# Author Correction: Primase promotes the competition between transcription and replication on the same template strand resulting in DNA damage

**DOI:** 10.1038/s41467-024-44904-0

**Published:** 2024-01-15

**Authors:** Weifeng Zhang, Zhuo Yang, Wenjie Wang, Qianwen Sun

**Affiliations:** 1 Center for Plant Biology, School of Life Sciences, Tsinghua University, 100084 Beijing, China; 2 Tsinghua-Peking Center for Life Sciences, 100084 Beijing, China

**Keywords:** Leaf development, Genomic instability, DNA replication

Correction to: *Nature Communications* 10.1038/s41467-023-44443-0, published online 02 January 2024

In the Acknowledgements section of this article, the grant numbers relating to the National Natural Science Foundation of China given for Qianwen Sun were incorrectly given as ‘31822028 and 91940306’ and should have been ‘32261133529 and 32170321’. The original article has been corrected.

