# Supplementary material for: Primase promotes the competition between transcription and replication on the same template strand resulting in DNA damage
Source: Nat Commun. 2024 Jan 2;15:73. doi: 10.1038/s41467-023-44443-0 (PMC10761990; doi:10.1038/s41467-023-44443-0)
Supplement: Supplementary file 1 — Supplementary Information [file 41467_2023_44443_MOESM1_ESM.pdf]

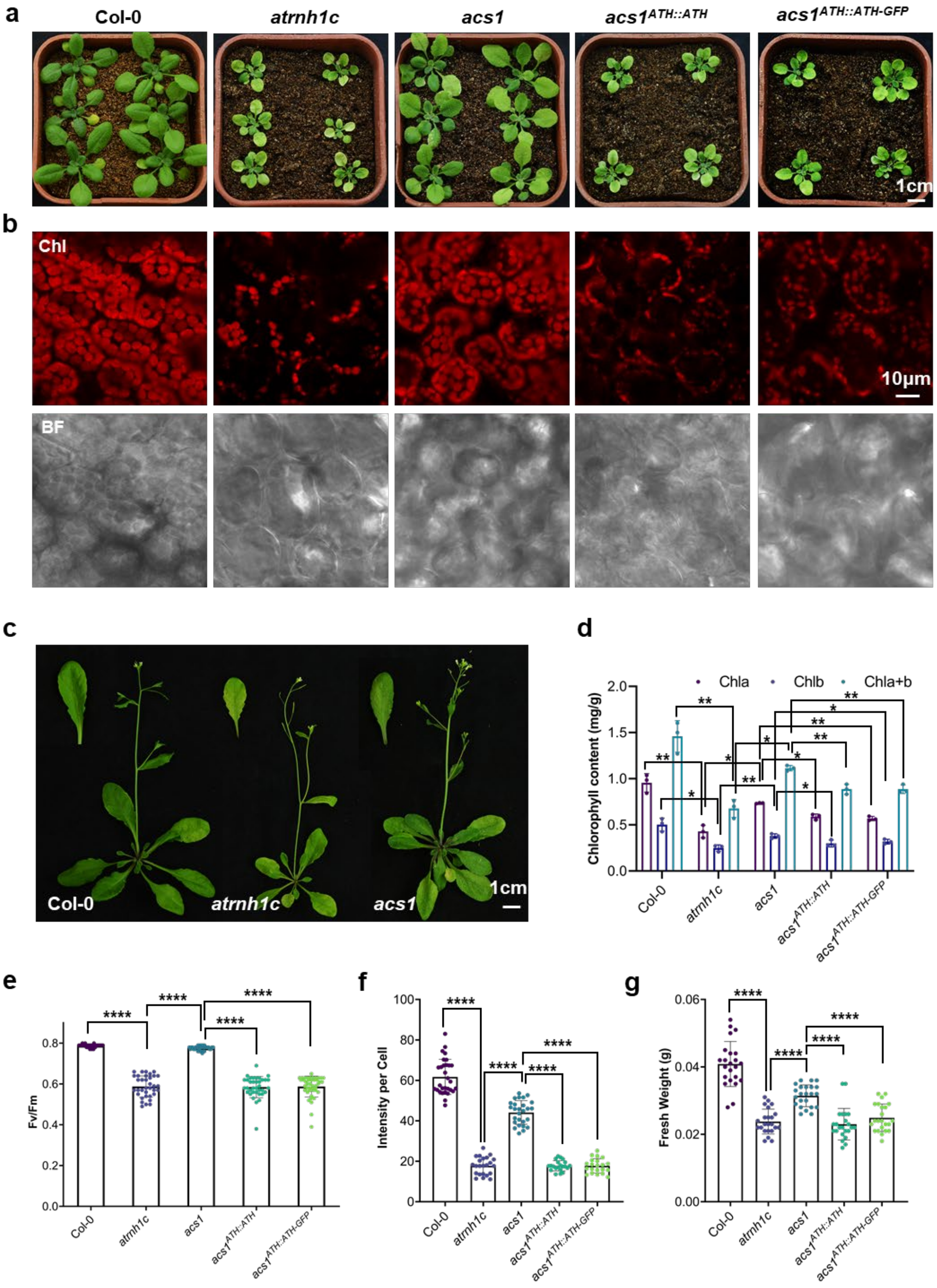

Supplementary Figure 1. Phenotypic characterization of the *acs1* mutant.

(**a**) The phenotypes of 30-day-old Col-0, *atrnh1c*, *acs1*, *acs1*$^{ATH::ATH}$, and *acs1*$^{ATH::ATH\text{-}GFP}$ plants.

Scale bar, 1 cm. (**b**) Cytological observation of chloroplasts in the leaves of the plants indicated in (**a**). Chloroplasts were distinguished by the chlorophyll autofluorescence (Chl, red). BF, bright field. Scale bar, 10 μm. (**c**) The phenotypes of Col-0, *atrnh1c*, *acs1*, *acs1*$^{ATH::ATH}$, and *acs1*$^{ATH::ATH\text{-}GFP}$ plants at the flowering stage (45-day-old). Representative rosette leaves are shown in the upper right of corresponding plants. Scale bar, 1 cm. (**d**) The chlorophyll contents of 21-day-old Col-0, *atrnh1c*, *acs1*, *acs1*$^{ATH::ATH}$, and *acs1*$^{ATH::ATH\text{-}GFP}$ plant leaves. Three biological replicates were performed. The graphs represent the mean ± SD. *P < 0.05; **P < 0.01 by unpaired two-sided t test. (**e**) Chlorophyll Fv/Fm values of 21-day-old Col-0, *atrnh1c*, *acs1*, *acs1*$^{ATH::ATH}$, and *acs1*$^{ATH::ATH\text{-}GFP}$ plants. Data were calculated from 35 plants, indicated by individual dots. The graphs represent the mean ± SD. ****P < 0.0001 by unpaired two-sided t test. (**f**) The intensity of chlorophyll autofluorescence in leaf cells of different plants is indicated in (**b**). Data were calculated from at least 20 cells, indicated by individual dots. The graphs represent the mean ± SD. ****P < 0.0001 by unpaired two-sided t test. (**g**) Fresh weight of 21-day-old Col-0, *atrnh1c*, *acs1*, *acs1*$^{ATH::ATH}$, and *acs1*$^{ATH::ATH\text{-}GFP}$ plants. Data were calculated from 23 plants, indicated by individual dots. The graphs represent the mean ± SD. ****P < 0.0001 by unpaired two-sided t test. Source data are provided as a Source Data file.

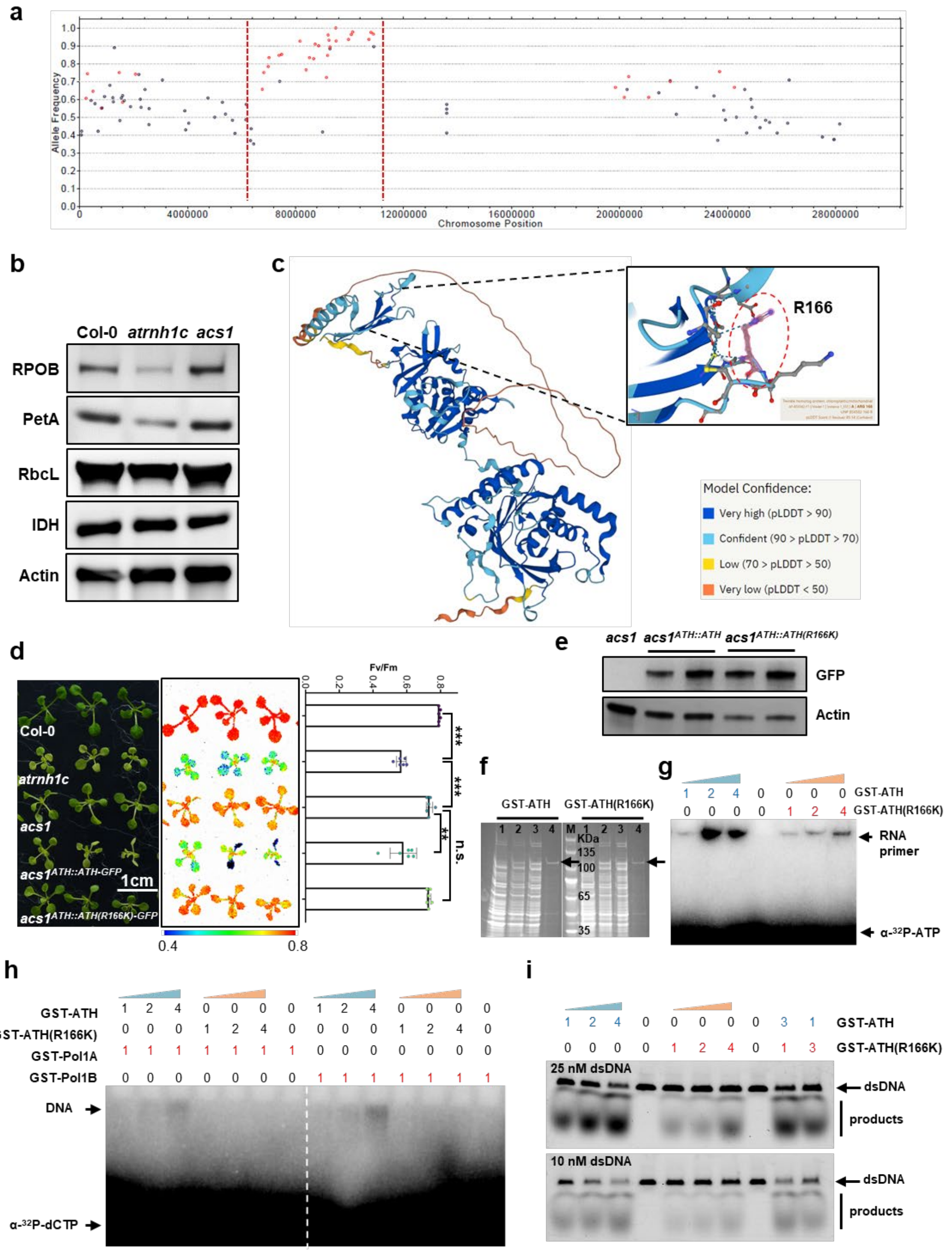

Supplementary Figure 2. The R166K mutation in ATH is responsible for phenotypic recovery in *acs1*.

(**a**) SHORE-map analysis mapped a region in chromosome 1 that is highly correlated with

phenotypic variation. (**b**) Immunoblot analysis of chloroplast proteins RPOB, PetA, and RbcL, and mitochondrial protein IDH in Col-0, *atrnh1c*, and *acs1*, respectively. (**c**) ATH protein structure prediction by Alphafold. The position of the R166 amino acid changed in the *acs1* mutant is shown in the right panel. (**d**) Photographs and chlorophyll fluorescence images of 14-day-old Col-0, *atrnh1c*, *acs1*, *acs1*$^{ATH::ATH\text{-}GFP}$, and *acs1*$^{ATH::ATH(R166K)\text{-}GFP}$ plants. Chlorophyll Fv/Fm values are presented in the right panel. Scale bar, 1 cm. Data were calculated from 6 plants, indicated by individual dots. The graphs represent the mean ± SD. Statistical testing was performed using by unpaired two-sided t test. **P < 0.01; ***P < 0.001; n.s, not significant. (**e**) Immunoblot analysis of ATH-GFP or ATH(R166K)-GFP levels in transgenic plants. (**f**) SDS-PAGE analysis of expression and purification of GST-ATH and GST-ATHR166K proteins. lane 1: cell lysate before induction, lane 2: cell lysate after 16 h of induction by 0.5 mM IPTG at 18°C, lane 3: supernatant after sonication and centrifugation, lane 4: purified protein eluted by 50 mM reduced glutathione. The predicted size of GST-ATH proteins was indicated by arrows. (**g**) Oligoribonucleotide synthesis by wild type ATH and ATH(R166K) using a ssDNA template containing the sequence 5'-$(T)_7$GGGA$(T)_7$-3'. Proteins were present from 100 to 400 nM as indicated. Each reaction was labeled with [α-^{32}P]-ATP. (**h**) RNA-dependent DNA synthesis using a ssDNA template containing the sequence 5'-$(T)_7$A-$(T)_6$GGGGA$(T)_9$-3'. Wild type ATH and ATH(R166K) were used to prime plant organellar DNA polymerases Pol1A and Pol1B. Coupled primase-DNA polymerase reactions were labeled with [α-^{32}P]-dCTP. Pol1A and Pol1B were present at 100 nM, ATH and ATH(R166K) were from 100 to 400 nM as indicated. (**i**) Helicase assay of the GST-ATH and GST-ATHR166K proteins. 25 nM (up panel) and 10 nM (down panel) 3'-FAM-tagged dsDNA were used as substrate, respectively. Both proteins were loaded from 100 to 400 nM as indicated. Source data are provided as a Source Data file.

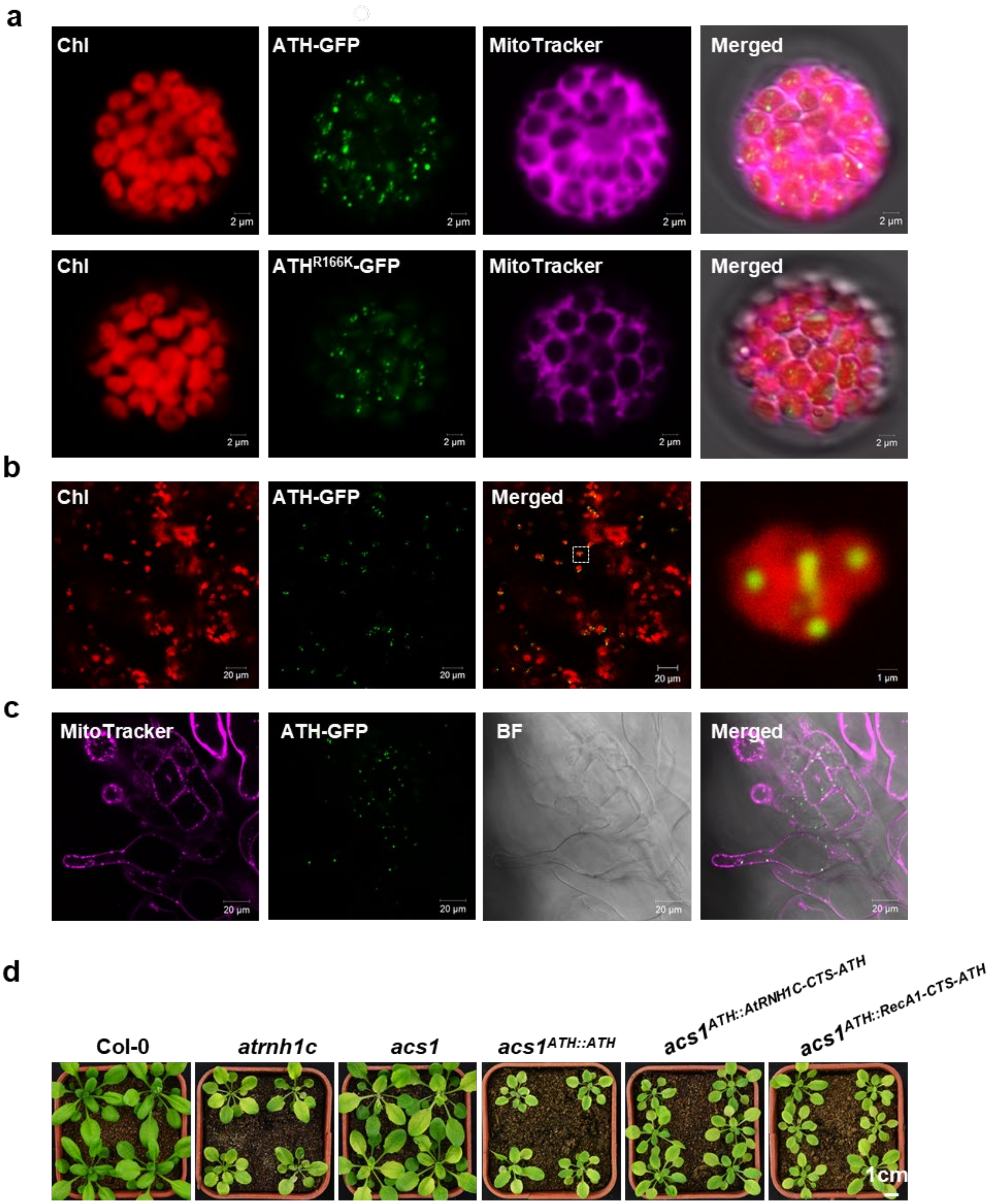

Supplementary Figure 3. Subcellular localization of ATH protein.

(**a**) Subcellular localization of GFP fused to the full-length ATH or ATH(R166K) protein in Arabidopsis protoplasts. Chl, chlorophyll autofluorescence (red); ATH-GFP, ATH fused with GFP (green); MitoTracker, mitochondria marker (Magenta). Scale bars, 2 μm. (**b**) Subcellular localization

of ATH-GFP in tobacco leaves. The right panel is the zoom-in image of the boxed area on left. Scale bars, 20 μm in the left panels and 1 μm in the right magnified image. (**c**) Subcellular localization of GFP fused to the full-length ATH protein in the roots of transgenic plants. MitoTracker, mitochondria marker (Magenta); ATH-GFP, ATH fused with GFP (green); BF, bright field. Scale bars, 20 μm. (**d**) The phenotypes of 30-day-old Col-0, *atrnh1c*, *acs1*, *acs1*$^{ATH::ATH}$, *acs1*$^{ATH::AtRNH1C\text{-}CTS\text{-}ATH}$, and *acs1*$^{ATH::RecA1\text{-}CTS\text{-}ATH}$ plants. Scale bar, 1 cm.

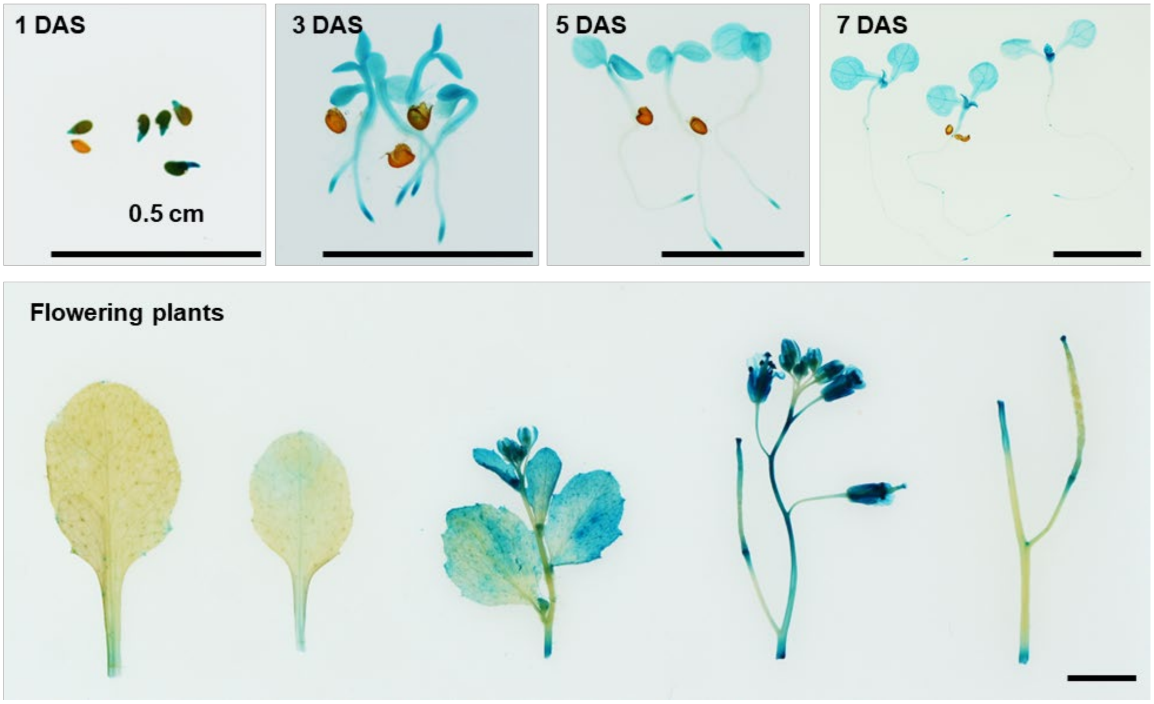

Supplementary Figure 4. Expression pattern of ATH during development.

Expression patterns of ATH-GUS in *acs1*$^{ATH::ATH\text{-}GUS}$ transformed Arabidopsis plants at different growth stages and tissues. DAS, days after sowing. Scale bars, 0.5 cm.

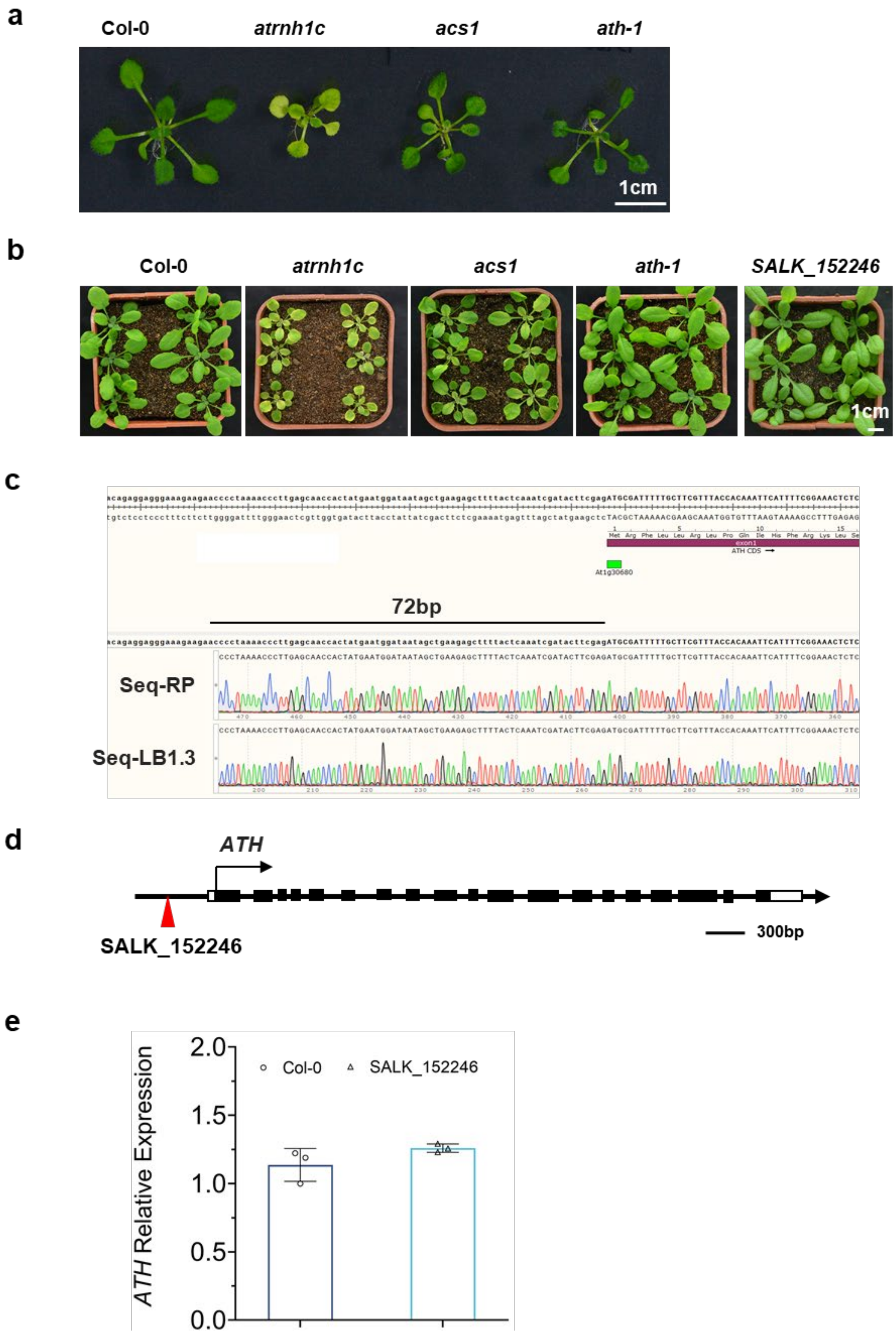

Supplementary Figure 5. Analysis of *ATH* T-DNA insertion line SALK_152246.

(**a**) The phenotypes of 18-day-old Col-0, *atrnh1c*, *acs1*, and *ath-1* plants. Scale bar, 1 cm. (**b**) The phenotypes of 30-day-old Col-0, *atrnh1c*, *acs1*, *ath-1*, and SALK_152246 plants. Scale bar, 1 cm. (**c**) Sequencing analysis of T-DNA insertion in SALK_152246 plants. (**d**) Schematic representations of the *ATH* gene and the insert position of T-DNA in SALK_152246 plants. (**e**) RT-qPCR analysis of *ATH* expression levels in SALK_152246 plants. Three biological replicates were performed. The graphs represent the mean ± SD. Source data are provided as a Source Data file.

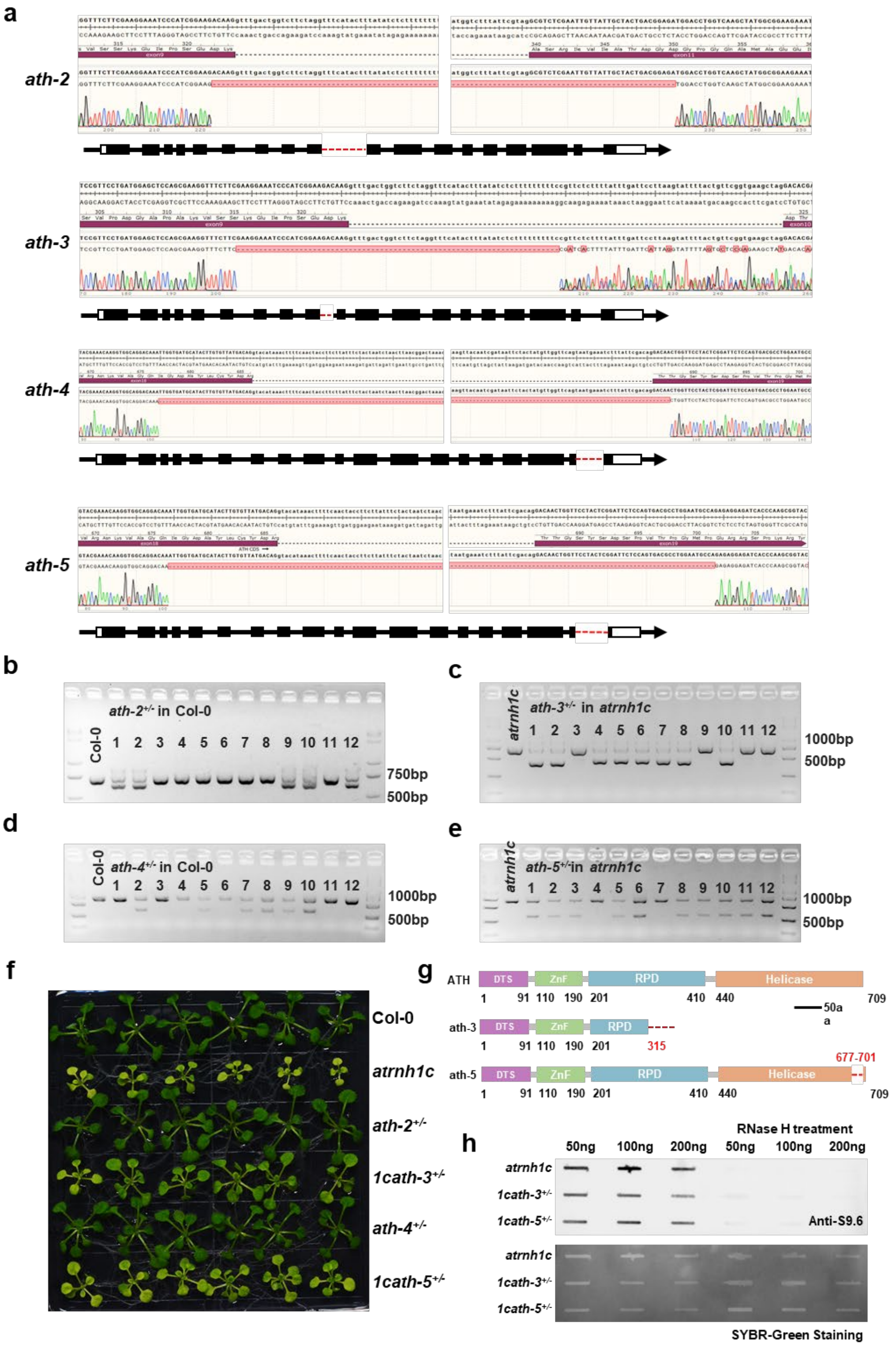

Supplementary Figure 6. Analysis of CRISPR/Cas9-mediated *ATH* genomic DNA deletion plants.

(**a**) Sequencing analysis of CRISPR/Cas9-mediated *ATH* genomic DNA deletion plants. (**b**) to (**e**) Genotyping of CRISPR/Cas9-mediated *ATH* genomic DNA deletion plants. (**f**) Phenotype of 14-day-old Col-0, *atrnh1c*, and heterozygous ATH mutants. (**g**) Schematic representations of the ATH proteins caused by CRISPR/Cas9-generated *ath-3* and *ath-5* mutants. (**h**) Slot-blot assays of the overall chloroplast R-loop levels of 21-day-old *atrnh1c*, *1cath-3*$^{+/-}$, and *1cath-5*$^{+/-}$ plants. RNase H-treated DNA was used as the negative control. R-loops were detected using the RNA:DNA hybrid antibody S9.6 (up panel). DNA loading was stained using SYBR Green (down panel).

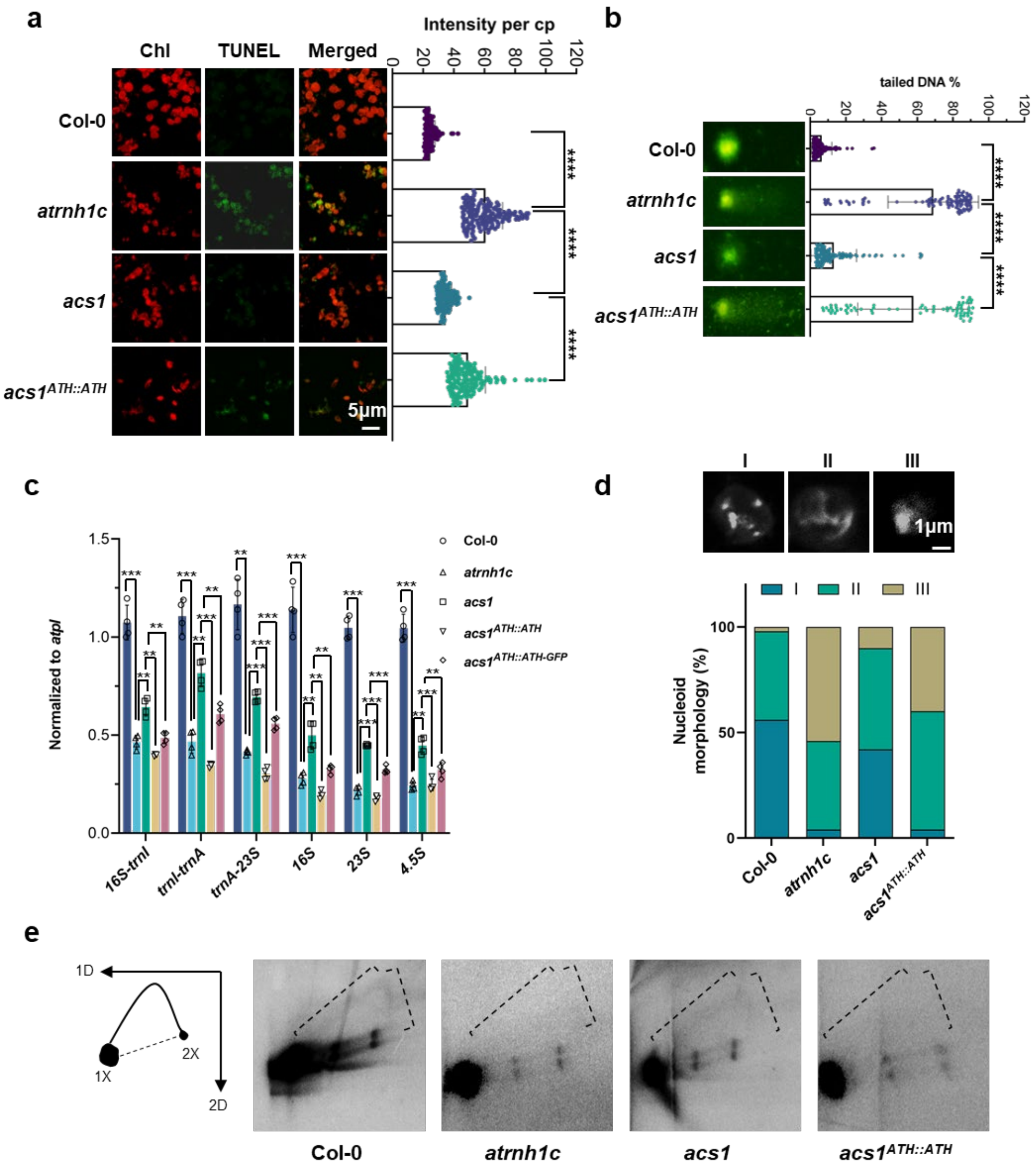

Supplementary Figure 7. *acs1* restricts DNA damage and rescues HO-TRCs competition in *atrnh1c*.

(**a**) TUNEL assays were used to detect DNA damage. At least 143 chloroplasts were analyzed for each genotype. ****, P < 0.0001 by unpaired two-sided t test. Scale bar, 5 μm. (**b**) Comet assay analysis of genome integrity in Col-0, *atrnh1c*, *acs1*, and *acs1*$^{ATH::ATH}$ chloroplasts. The neutral comet assay images of each sample are shown in the left panel. The right panel is the percentage of DNA in the comet tail versus the comet head (tailed DNA %). At least 70 chloroplasts were

analyzed for each genotype, indicated by an individual dot. ****, P < 0.0001 by unpaired two-sided t test. (**c**) RT-qPCR of nascent and mature chloroplast rRNA abundance. Expression levels were normalized to a chloroplast gene *atpI*. Four biological replicates were performed. The graphs represent the mean ± SD. **P < 0.01; ***P < 0.001 by unpaired two-sided t test. (**d**) Chloroplast nucleoids were stained with DAPI, and nucleoid states were categorized into three types (I, II, and III). 100 chloroplasts of each sample were used to calculate the fractions of nucleoid morphology. Scale bars, 1 µm. (**e**) Two-dimensional gel analysis detected DNA replication intermediates of 21-day-old Col-0, *atrnh1c*, *acs1*, and *acs1*$^{ATH::ATH}$ plants. The probe detects fragments in the chloroplast 16S rDNA region. DNA replication intermediates are illustrated in the left panel. Replication intermediates are indicated by dotted square brackets. Source data are provided as a Source Data file.

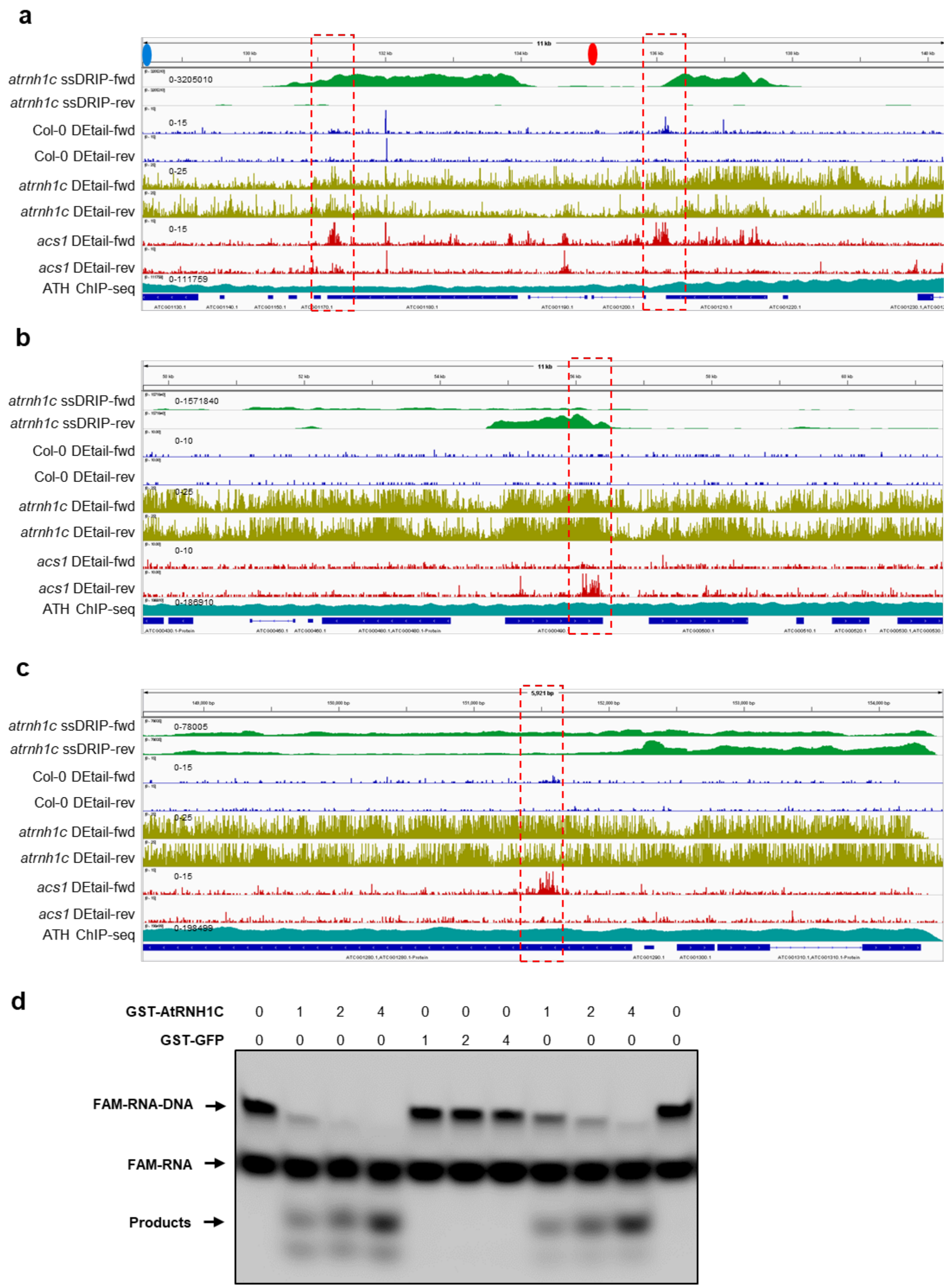

Supplementary Figure 8. Single-strand DNA damages are detected in the chloroplast genome.

(**a**) to (**c**) Snapshots of ssDRIP-seq, DEtail-seq, and ATH ChIP-seq in the regions where the HO-TRCs competition induces single-strand DNA damage of the competitive strand at the end of transcription units. Replicates and controls can be shown in GSE215443. The damaged sites are shown in red boxes. The red and blue ellipses show OriA and OriB. (**d**) In vitro RNase H activity analysis of AtRNH1C with RNA primer-like RNA:DNA hybrid (8-bp) as the substrate. The RNA:DNA hybrid substrate with FAM-labeled RNA (100 nM) was incubated with 100 to 400 nM purified GST-AtRNH1C or GST-GFP proteins for 30 min.

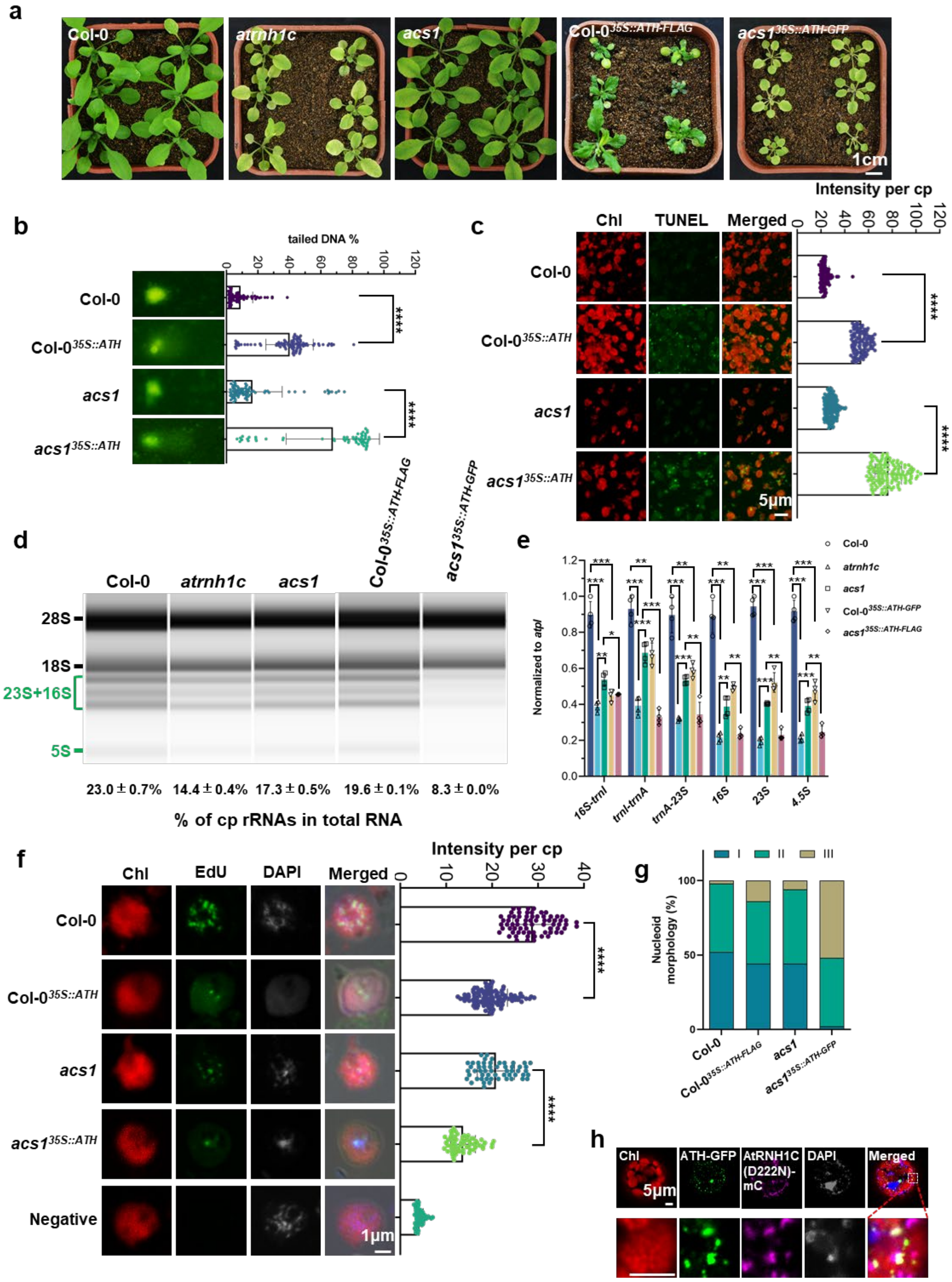

Supplementary Figure 9. Overexpression of ATH leads to aberrant plants and restrains transcription and replication in the chloroplast genome.

(**a**) The phenotype of 30-day-old plants of Col-0, *atrnh1c*, *acs1*, Col-0$^{35S::ATH\text{-}FLAG}$, and *acs1*$^{35S::ATH\text{-}GFP}$. Scale bar, 1 cm. (**b**) Comet assay analysis of Col-0, Col-0$^{35S::ATH\text{-}FLAG}$, *acs1*, and *acs1*$^{35S::ATH\text{-}GFP}$ chloroplasts. The left panel shows neutral comet assay images of each sample. The right panel is the percentage of DNA in the comet tail versus the comet head (tailed DNA %). At least 58 chloroplasts were analyzed for each genotype, and each chloroplast is indicated by an individual dot. The graphs represent the mean ± SD. ****, P < 0.0001 by unpaired two-sided t test. (**c**) TUNEL assays detect DNA damage. At least 109 chloroplasts were analyzed for each genotype. Scale bar, 5 μm. The graphs represent the mean ± SD. ****, P < 0.0001 by unpaired two-sided t test. (**d**) Bioanalyzer (Agilent 4200) analysis of total RNAs isolated from 21-day-old plant leaves. The calculation of the percentage of cp-rRNAs in total RNAs is shown at the bottom. (**e**) RT-qPCR of nascent and mature chloroplast rRNA abundance. Expression levels were normalized to a chloroplast gene *atpI*. Four biological replicates were performed. Graphs represent mean ± SD. *P < 0.05; **P < 0.01; ***P < 0.001 by unpaired two-sided t test. (**f**) EdU labeling detects the DNA replication intensity in chloroplasts. Representative images are shown on the left. Scale bar, 1 μm. DNA replication intensity was calculated from at least 55 chloroplasts, indicated by individual dots. The graphs represent the mean ± SD. ****P < 0.0001 by unpaired two-sided t test. (**g**) Chloroplast nucleoids were stained with DAPI, and nucleoid states were categorized into three types (**I**, **II**, and **III**). 100 chloroplasts of each sample were used to calculate the fractions of nucleoid morphology. (**h**) Co-localization of ATH-GFP and AtRNH1C(D222N)-mC (mCherry) in Col-0 protoplast. The panels beneath are the magnified images of the boxed areas; scale bars, 5 μm. Source data are provided as a Source Data file.

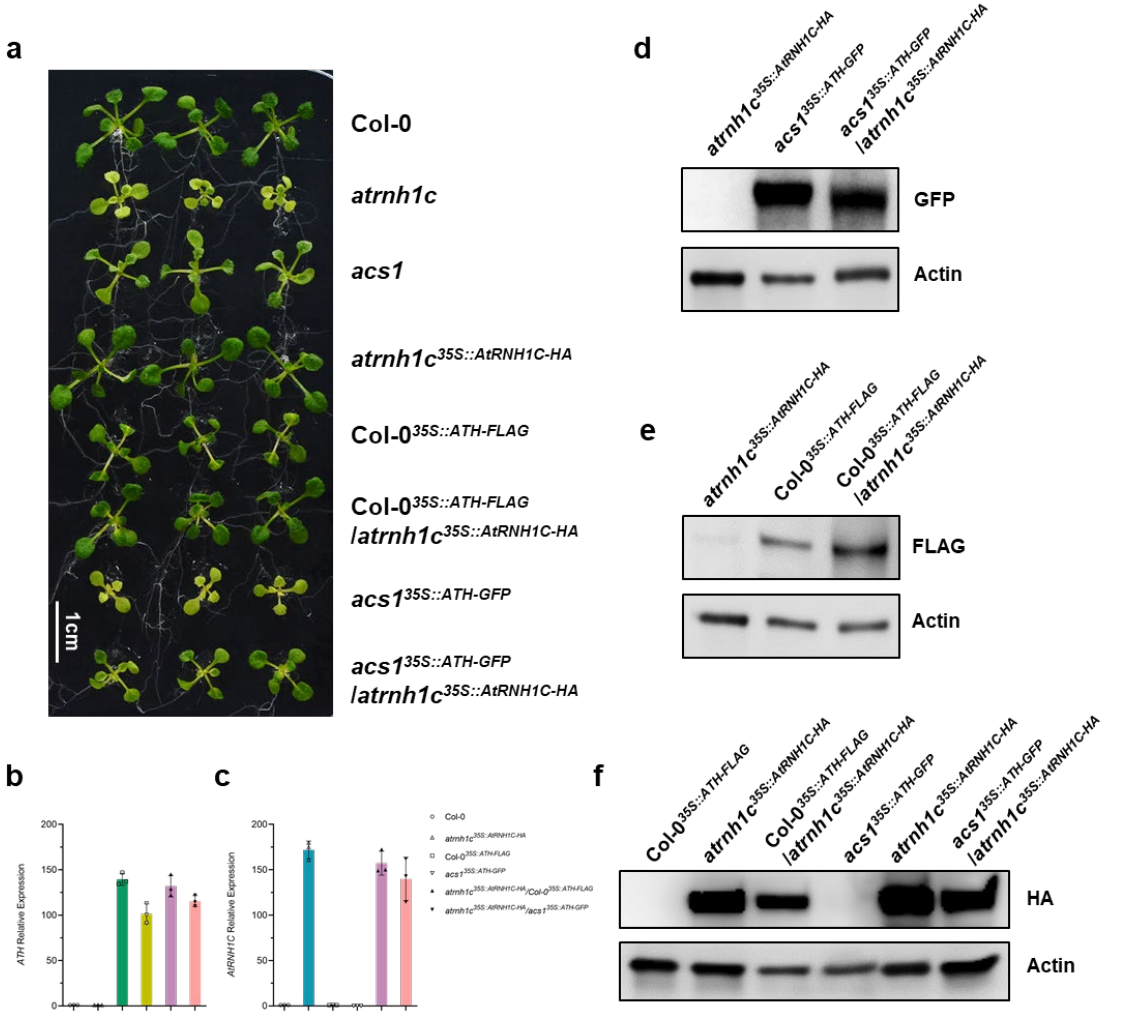

Supplementary Figure 10. Overexpression of AtRNH1C alleviates the aberrant phenotype of ATH overexpressing plants.

(**a**) The phenotypes of 14-day-old Col-0, *atrnh1c*, *acs1*, $atrnh1c^{35S::AtRNH1C\text{-}HA}$, $\text{Col-0}^{35S::ATH\text{-}FLAG}$, $\text{Col-0}^{35S::ATH\text{-}FLAG}/atrnh1c^{35S::AtRNH1C\text{-}HA}$ F_1, $acs1^{35S::ATH\text{-}GFP}$, and $acs1^{35S::ATH\text{-}GFP}/atrnh1c^{35S::AtRNH1C\text{-}HA}$ F_1 plants. Scale bar, 1 cm. (**b**) and (**c**) RT-qPCR analysis of *ATH* and *AtRNH1C* expression levels. Three biological replicates were performed. The graphs represent the mean ± SD. (**d**), (**e**) and (**f**) Immunoblot analysis of ATH-GFP (**d**), ATH-FLAG (**e**), and AtRNH1C-HA (**f**) protein levels in corresponding plants. Source data are provided as a Source Data file.

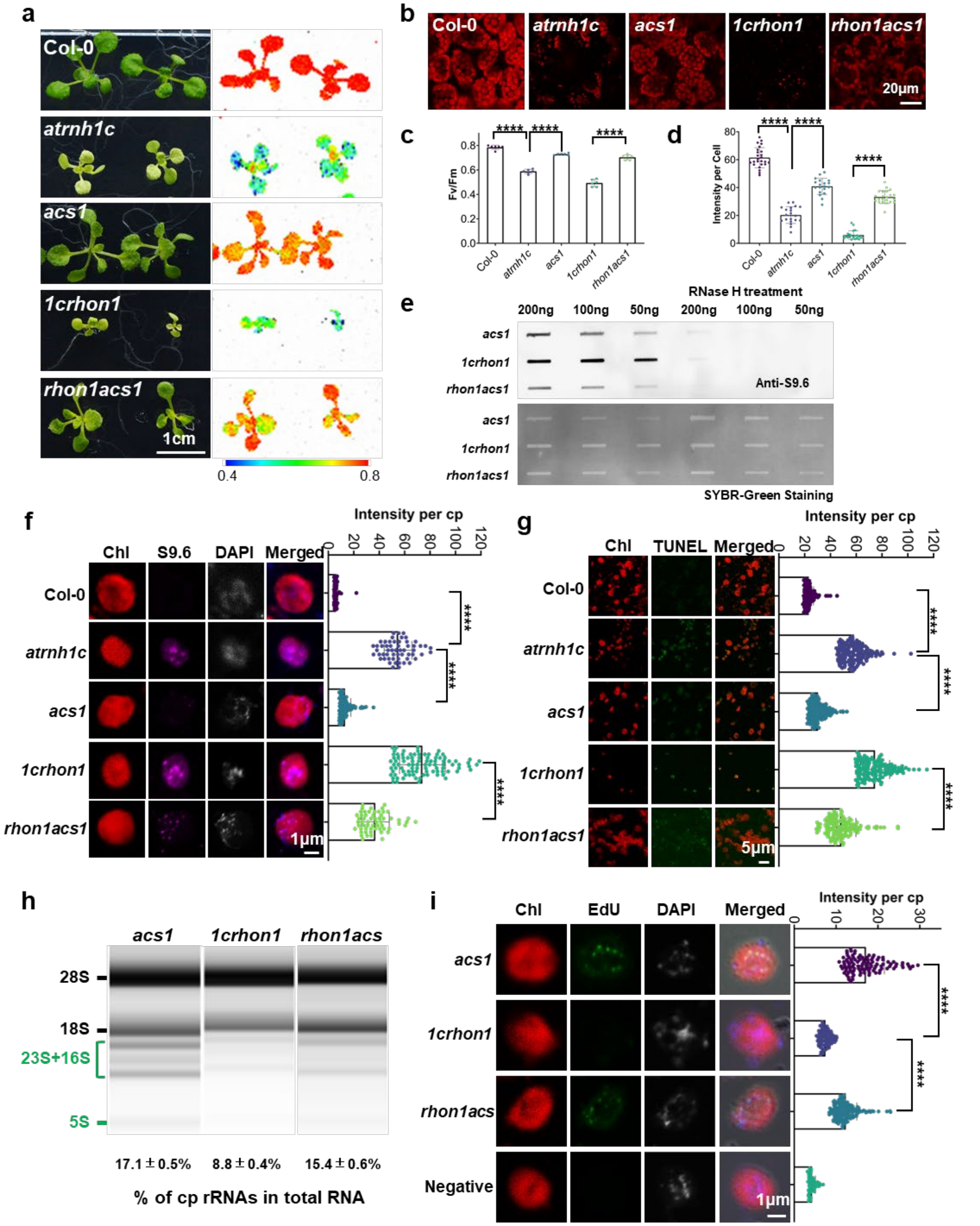

Supplementary Figure 11. *acs1* can rescue the deficiency of *1crhon1* by restricting HO-TRCs.

(**a**) Photographs and chlorophyll fluorescence images of 14-day-old Col-0, *atrnh1c*, *acs1*, *1crhon1*, and *rhon1acs1* plants. The color scale representing Fv/Fm is given at the bottom of the right panel.

Scale bar, 1 cm. (**b**) Cytological observation of chloroplasts in the leaves of 21-day-old plants. Chloroplasts were distinguished by chlorophyll autofluorescence (red). Scale bar, 20 μm. (**c**) Chlorophyll Fv/Fm values of plants are indicated in (**a**). Data were calculated from 6 plants, indicated by individual dots. ****P < 0.0001 by unpaired two-sided t test. (**d**) The intensity of chlorophyll autofluorescence per leaf cell of different plants. Data were calculated from at least 19 cells, indicated by individual dots. The graphs represent the mean ± SD. ****P < 0.0001 by unpaired two-sided t test. (**e**) Slot-blot assays of the overall chloroplast R-loop levels of 21-day-old plants. RNase H-treated DNA was used as the negative control. (**f**) R-loop signal (magenta) accumulation in chloroplasts was detected by S9.6 immunostaining. Representative images are shown on the left. R-loop signal intensity was calculated from at least 56 chloroplasts, indicated by individual dots. ****P < 0.0001 by unpaired two-sided t test. Scale bar, 1 μm. (**g**) TUNEL assays were used to detect DNA damage. At least 144 chloroplasts were analyzed for each genotype. ****P < 0.0001 by unpaired two-sided t test. Scale bar, 5 μm. (**h**) Bioanalyzer (Agilent 4200) results showing total RNAs isolated from 21-day-old plant leaves. The calculation of the percentage of cp-rRNAs in total RNAs is shown at the bottom. (**i**) Measurement of the DNA replication intensity in chloroplasts by EdU labeling. Representative images are shown on the left. Scale bar, 1 μm. DNA replication intensity was calculated from at least 70 chloroplasts, indicated by individual dots. The graphs represent the mean ± SD. ****P < 0.0001 by unpaired two-sided t test. Source data are provided as a Source Data file.

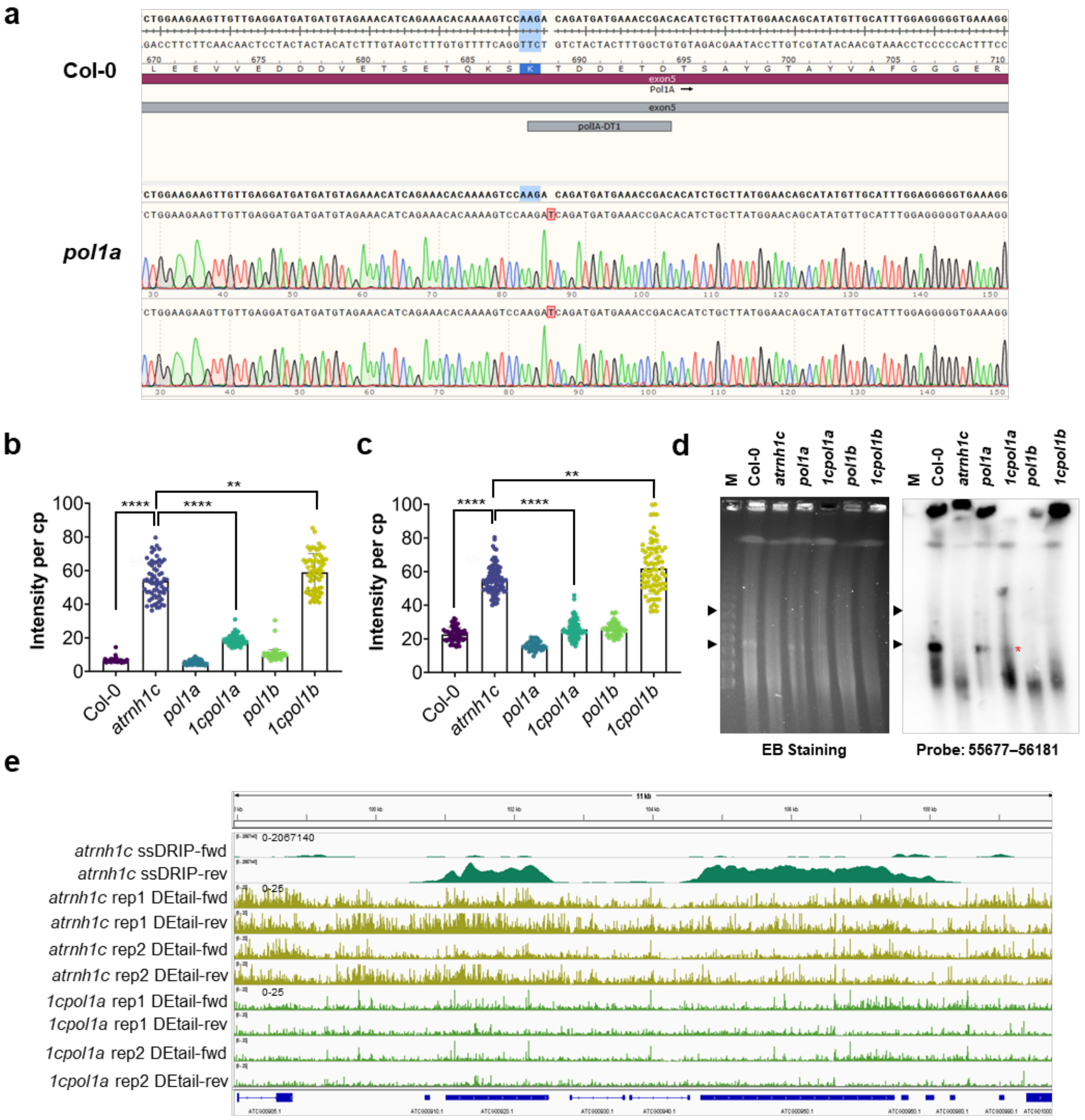

Supplementary Figure 12. *Pol1a* rescues the deficiency of *atrnh1c* by restricting HO-TRCs in chloroplasts.

(**a**) Sequencing analysis of CRISPR/Cas9-mediated *Pol1A* genomic DNA mutation plants. (**b**) R-loop signal intensity in chloroplasts detected by S9.6 immunostaining. Data were calculated from at least 60 chloroplasts, indicated by individual dots. The graphs represent the mean ± SD. **P < 0.01; ****P < 0.0001 by unpaired two-sided t test. (**c**) TUNEL signal intensity in chloroplasts. Data were calculated from at least 40 chloroplasts, indicated by individual dots. The graphs represent the mean ± SD. **P < 0.01; ****P < 0.0001 by unpaired two-sided t test. (**d**) PFGE assay of cpDNA from 21-day-old Col-0, *atrnh1c*, *pol1a*, *1cpol1a*, *pol1b*, and *1cpol1b* plants. The left panel shows

ethidium bromide staining, and the right panel shows blot hybridization of probe 55677–56181 (a 505-bp *rbcL* gene fragment). A Lambda Ladder (New England Biolabs; N0341) was used to indicate the molecular weight. Arrowheads indicate the structures of cpDNA monomers and dimers. The red asterisk shows the rescue of cpDNA monomers in *1cpol1a* compared to *atrnh1c*. (**e**) Snapshots of DEtail-seq in the rDNA region of the chloroplast of 21-day-old *atrnh1c* and *1cpol1a* plants. Source data are provided as a Source Data file.
