# Supplementary material for: Primase promotes the competition between transcription and replication on the same template strand resulting in DNA damage
Source: Nat Commun. 2024 Jan 2;15:73. doi: 10.1038/s41467-023-44443-0 (PMC10761990; doi:10.1038/s41467-023-44443-0)
Supplement: Supplementary file 3 — Description of Additional Supplementary Files [file 41467_2023_44443_MOESM3_ESM.pdf]

Description of Additional Supplementary Files

File Name: Supplementary Data 1
Description: List of primers and oligonucleotides used in this study.
