# Supplementary material for: Primase promotes the competition between transcription and replication on the same template strand resulting in DNA damage
Source: Nat Commun. 2024 Jan 2;15:73. doi: 10.1038/s41467-023-44443-0 (PMC10761990; doi:10.1038/s41467-023-44443-0)
Supplement: Supplementary file 5 — Reporting Summary [file 41467_2023_44443_MOESM5_ESM.pdf]

nature portfolio

Corresponding author(s): Qianwen Sun

Last updated by author(s): Dec 8, 2023

Reporting Summary

Nature Portfolio wishes to improve the reproducibility of the work that we publish. This form provides structure for consistency and transparency in reporting. For further information on Nature Portfolio policies, see our Editorial Policies and the Editorial Policy Checklist.

Statistics

For all statistical analyses, confirm that the following items are present in the figure legend, table legend, main text, or Methods section.

n/a	Confirmed	
☐	☒	The exact sample size (*n*) for each experimental group/condition, given as a discrete number and unit of measurement
☐	☒	A statement on whether measurements were taken from distinct samples or whether the same sample was measured repeatedly
☐	☒	The statistical test(s) used AND whether they are one- or two-sided<br>*Only common tests should be described solely by name; describe more complex techniques in the Methods section.*
☒	☐	A description of all covariates tested
☐	☒	A description of any assumptions or corrections, such as tests of normality and adjustment for multiple comparisons
☐	☒	A full description of the statistical parameters including central tendency (e.g. means) or other basic estimates (e.g. regression coefficient) AND variation (e.g. standard deviation) or associated estimates of uncertainty (e.g. confidence intervals)
☐	☒	For null hypothesis testing, the test statistic (e.g. *F*, *t*, *r*) with confidence intervals, effect sizes, degrees of freedom and *P* value noted<br>*Give P values as exact values whenever suitable.*
☒	☐	For Bayesian analysis, information on the choice of priors and Markov chain Monte Carlo settings
☒	☐	For hierarchical and complex designs, identification of the appropriate level for tests and full reporting of outcomes
☒	☐	Estimates of effect sizes (e.g. Cohen's *d*, Pearson's *r*), indicating how they were calculated

Our web collection on statistics for biologists contains articles on many of the points above.

Software and code

Policy information about availability of computer code

Data collection	ZEN blue edition (version 3.4) was used for confocal imaging by ZEISS LSM880, Roche LightCycler480 was used for qPCR, GE ImageQuant LAS 4000 was used for western-blot imaging, bowtie2 (version 2.3.5.1) and fastqc (version 0.11.7) were used for ChIP-seq .
Data analysis	GraphPad Prism 9, Microsoft Excel 2019, Image J 1.52, deeptools (version 3.5.1), NGPhylogeny.fr, iTOL (version 5)

For manuscripts utilizing custom algorithms or software that are central to the research but not yet described in published literature, software must be made available to editors and reviewers. We strongly encourage code deposition in a community repository (e.g. GitHub). See the Nature Portfolio guidelines for submitting code & software for further information.

Data

Policy information about availability of data

All manuscripts must include a data availability statement. This statement should provide the following information, where applicable:

- Accession codes, unique identifiers, or web links for publicly available datasets
- A description of any restrictions on data availability
- For clinical datasets or third party data, please ensure that the statement adheres to our policy

The sequencing data generated in this study have been deposited into Gene Expression Omnibus (GEO) with the accession number GSE215443(https://www.ncbi.nlm.nih.gov/geo/query/acc.cgi?acc=GSE215443). Source data are provided with this paper.

Research involving human participants, their data, or biological material

Policy information about studies with human participants or human data. See also policy information about sex, gender (identity/presentation), and sexual orientation and race, ethnicity and racism.

Reporting on sex and gender	N/A
Reporting on race, ethnicity, or other socially relevant groupings	N/A
Population characteristics	N/A
Recruitment	N/A
Ethics oversight	N/A

Note that full information on the approval of the study protocol must also be provided in the manuscript.

Field-specific reporting

Please select the one below that is the best fit for your research. If you are not sure, read the appropriate sections before making your selection.

☒ Life sciences ☐ Behavioural & social sciences ☐ Ecological, evolutionary & environmental sciences

For a reference copy of the document with all sections, see nature.com/documents/nr-reporting-summary-flat.pdf

Life sciences study design

All studies must disclose on these points even when the disclosure is negative.

Sample size	Sample size of each experiment are indicated in the figure legends.
Data exclusions	No data excluded.
Replication	Experiments were repeated three times with similar results as indicated in the figure legends.
Randomization	plant materials were grown in the same conditions and collected randomly without any bias.
Blinding	Blinding was not applied during the experiments since the same experimental procedure and analysis pipelines were preformed in parallel for samples with different genetics backgrounds in each experimental group.

Reporting for specific materials, systems and methods

We require information from authors about some types of materials, experimental systems and methods used in many studies. Here, indicate whether each material, system or method listed is relevant to your study. If you are not sure if a list item applies to your research, read the appropriate section before selecting a response.

Materials & experimental systems

n/a	Involved in the study	
☐	☒	Antibodies
☒	☐	Eukaryotic cell lines
☒	☐	Palaeontology and archaeology
☒	☐	Animals and other organisms
☒	☐	Clinical data
☒	☐	Dual use research of concern
☐	☒	Plants

Methods

n/a	Involved in the study	
☐	☒	ChIP-seq
☒	☐	Flow cytometry
☒	☐	MRI-based neuroimaging

Antibodies

Antibodies used	S9.6 antibody: 10 μg for DRIP, 1:100 for IF, 1:1000 for slot blot. GFP antibody (abcam, ab290): 10 μg for ChIP. Anti-FLAG (Sigma, F1804): 1:5000 dilution for western blot. anti-GFP (ABclonal, AE012): 1:5000 dilution for western blot. anti-HA (Beyotime, AF5057): 1:5000 dilution for western blot. anti-plant-actin (ABclonal, AC009): 1:5000 dilution for western blot. anti-RPOB (PhytoAB, PHY1701):

1:5000 dilution for western blot. anti-PetA (PhytoAB, PHY0023): 1:5000 dilution for western blot. anti-RbcL (Agrisera, AS03037A): 1:5000 dilution for western blot. anti-PsaA (PhytoAB, PHY0053A): 1:5000 dilution for western blot. anti-IDH1 (PhytoAB, PHY0098A): 1:5000 dilution for western blot. Goat Anti-Mouse-HRP (Easybio, BE0102): 1:5000 dilution for western blot. Goat Anti-Rabbit-HRP (Easybio, BE0101): 1:5000 dilution for western blot.

Validation

All the antibodies used in this study have been cited or published, and are commercially available:
https://www.abcam.cn/products%2fprimary-antibodies%2fgfp-antibody-ab290.html
https://www.sigmaaldrich.cn/CN/en/product/sigma/f1804
https://abclonal.com.cn/catalog/AE012
https://www.beyotime.com/product/AF5057.htm
https://abclonal.com.cn/catalog/AC009
https://www.phytoab.com/rpob%20antibody
https://www.phytoab.com/products/primary-antibodies/peta%20antibody
https://www.agrisera.com/en/artiklar/rbcl-rubisco-large-subunit-form-i-and-form-ii-10-l-2.html
https://www.phytoab.com/products/primary-antibodies/psaa%20antibody
https://www.phytoab.com/products/primary-antibodies/idh1%20antibody
http://bioeasytech.com/product/2901.html
http://bioeasytech.com/product/2907.html

Dual use research of concern

Policy information about dual use research of concern

Hazards

Could the accidental, deliberate or reckless misuse of agents or technologies generated in the work, or the application of information presented in the manuscript, pose a threat to:

No	Yes	
☒	☐	Public health
☒	☐	National security
☒	☐	Crops and/or livestock
☒	☐	Ecosystems
☒	☐	Any other significant area

Experiments of concern

Does the work involve any of these experiments of concern:

No	Yes	
☒	☐	Demonstrate how to render a vaccine ineffective
☒	☐	Confer resistance to therapeutically useful antibiotics or antiviral agents
☒	☐	Enhance the virulence of a pathogen or render a nonpathogen virulent
☒	☐	Increase transmissibility of a pathogen
☒	☐	Alter the host range of a pathogen
☒	☐	Enable evasion of diagnostic/detection modalities
☒	☐	Enable the weaponization of a biological agent or toxin
☒	☐	Any other potentially harmful combination of experiments and agents

Plants

Seed stocks	The T-DNA insertion mutants atrnh1c, rhon1, and SALK_152246 were obtained from Nottingham Arabidopsis Stock Centre, UK.
Novel plant genotypes	Arabidopsis transgenic line were generated by the floral-dip method using Agrobacterium tumefaciens GV3101.
Authentication	T-DNA insertion mutants were verified by PCR and sequencing, transgenic plants were verified by RT-qPCR and western blot.

ChIP-seq

Data deposition

☒ Confirm that both raw and final processed data have been deposited in a public database such as GEO.

☒ Confirm that you have deposited or provided access to graph files (e.g. BED files) for the called peaks.

Data access links<br>*May remain private before publication.*	https://www.ncbi.nlm.nih.gov/geo/query/acc.cgi?acc=GSE215443
Files in database submission	GSM6637218 ATH_ChIP_rep1<br>GSM6637219 ATH_ChIP_rep2<br>GSM6637220 GFP_ChIP_control
Genome browser session<br>(e.g. UCSC)	Processed and analysed genomic data has been deposited in GEO (https://www.ncbi.nlm.nih.gov/geo/query/acc.cgi?acc=GSE215443).

Methodology

Replicates	All ChIP-seq datasets have two biological replicates.
Sequencing depth	All samples were sequenced single end 50 with depth ranging between 10~13 million reads.
Antibodies	anti-GFP antibody (Abcam, ab290)
Peak calling parameters	No peaks were called in this work.
Data quality	Trim galore was used for filtering reads. Command used:<br>trim_galore --phred33 --fastqc --paired ${input} --gzip &
Software	bowtie2 (version 2.3.5.1), fastqc (version 0.11.7), deeptools (version 3.5.1)
